# Supplementary material for: Validation of the Rosenberg Self-Esteem Scale among the Iranian adult population: A cross-sectional study
Source: PLoS One. 2025 Dec 15;20(12):e0336969. doi: 10.1371/journal.pone.0336969 (PMC12799268; doi:10.1371/journal.pone.0336969)
Supplement: S1 Appendix — (DOCX) [file pone.0336969.s001.docx]

**S1 Appendix.** Persian version of the Rosenberg Self-Esteem Scale (RSES)

**Section 1: Demographic characteristics**

**Gender:** Male 🞎 / Female 🞎

**Age:** ....................

**Marital status:** Single 🞎 / Married 🞎 / Divorced 🞎 / Widowed 🞎

**Education level:** Primary 🞎 / Diploma 🞎 / Associate 🞎 / Bachelor's degree 🞎 / Master's degree 🞎 / Doctorate 🞎

**Employment status:** Self-employed 🞎 / Retired 🞎 / Employed 🞎 / Unemployed 🞎 / Student 🞎

**Section 2: Persian version of the Rosenberg Self-Esteem Scale (RSES)**

Dear participant,

The scale provided is intended to assess your self-esteem.

It consists of 10 items that will be scored based on a four-point scale ranging from "strongly agree" to "strongly disagree".

It will only take about two minutes of your valuable time to complete.

First, carefully study each item and then mark your desired answer in the box opposite it.

| **No.** | **Item** | **Strongly agree** | **Agree** | **Disagree** | **Strongly disagree** |
| --- | --- | --- | --- | --- | --- |
| **1** | I feel that I am a valuable person. (At least the same value as others) |  |  |  |  |
| **2** | I feel that I have good qualities. |  |  |  |  |
| **3** | All things considered, I usually feel like a failure. |  |  |  |  |
| **4** | I can do things as well as other people. |  |  |  |  |
| **5** | I feel like I don't have much to be proud of. |  |  |  |  |
| **6** | I have a positive attitude towards myself. |  |  |  |  |
| **7** | I am generally satisfied with myself. |  |  |  |  |
| **8** | I wish I could have more respect for myself. |  |  |  |  |
| **9** | Sometimes I feel useless. |  |  |  |  |
| **10** | Sometimes I think that I have no skills in any fields. |  |  |  |  |
