# Supplementary material for: Validation of the Rosenberg Self-Esteem Scale among the Iranian adult population: A cross-sectional study
Source: PLoS One. 2025 Dec 15;20(12):e0336969. doi: 10.1371/journal.pone.0336969 (PMC12799268; doi:10.1371/journal.pone.0336969)
Supplement: Attachment — Submitted filename: Response to reviewers.docx [file pone.0336969.s003.docx]

**Dear Editor, PLOS One,**

Thank you for carefully reviewing and considering our manuscript, titled **"Validity and reliability of the Rosenberg Self-Esteem Scale: A methodological study among the Iranian adult population"**, submitted to ***PLOS One***.

We sincerely appreciate the thoughtful comments and suggestions provided by the reviewers, which have greatly enhanced the quality and depth of our manuscript. In the revised version, all changes made in response to the feedback have been highlighted. Additionally, detailed responses to each comment are provided below.

Please feel free to contact me if you have any questions or need further clarification. We look forward to your response regarding the manuscript.

Thank you once again for your time and support.

Sincerely,

Corresponding author

| **Journal Requirements** | **Response** |
| --- | --- |
| Journal Requirements:  When submitting your revision, we need you to address these additional requirements.  1. Please ensure that your manuscript meets PLOS ONE's style requirements, including those for file naming. The PLOS ONE style templates can be found at  https://journals.plos.org/plosone/s/file?id=wjVg/PLOSOne_formatting_sample_main_body.pdf and  https://journals.plos.org/plosone/s/file?id=ba62/PLOSOne_formatting_sample_title_authors_affiliations.pdf  2. We note that your Data Availability Statement is currently as follows: [All relevant data are within the manuscript and its Supporting Information files.]  Please confirm at this time whether or not your submission contains all raw data required to replicate the results of your study. Authors must share the “minimal data set” for their submission. PLOS defines the minimal data set to consist of the data required to replicate all study findings reported in the article, as well as related metadata and methods (https://journals.plos.org/plosone/s/data-availability#loc-minimal-data-set-definition).  For example, authors should submit the following data:  - The values behind the means, standard deviations and other measures reported;  - The values used to build graphs;  - The points extracted from images for analysis.  Authors do not need to submit their entire data set if only a portion of the data was used in the reported study.  If your submission does not contain these data, please either upload them as Supporting Information files or deposit them to a stable, public repository and provide us with the relevant URLs, DOIs, or accession numbers. For a list of recommended repositories, please see https://journals.plos.org/plosone/s/recommended-repositories.  If there are ethical or legal restrictions on sharing a de-identified data set, please explain them in detail (e.g., data contain potentially sensitive information, data are owned by a third-party organization, etc.) and who has imposed them (e.g., an ethics committee). Please also provide contact information for a data access committee, ethics committee, or other institutional body to which data requests may be sent. If data are owned by a third party, please indicate how others may request data access. | Thank you so much for your support.  1. We ensured that our manuscript meets PLOS ONEs style requirements.  2. We added a “Data Availability Statement” section and provided information based on your recommendation. We uploaded our final data about the Persian version of the scale as Supporting Information File. |
| **Additional Editor Comments** | **Response** |
| This study examines the psychometric properties of the Persian Rosenberg Self-Esteem Scale (RSES) in Iranian adults, revealing a stable two-factor structure (PSE/NSE). However, several significant methodological concerns diminish its reliability:  1- Restricted Generalizability: The sample predominantly consists of young, single students, failing to represent the diverse population of Iran. | Thank you for your constructive feedback. We fully agree with you and have included these as limitations at the end of the discussion section. |
| 2- Deficiencies in factor analysis: EFA and CFA likely utilized the same sample, potentially resulting in overfitting, and items with low AVE values (<0.5) lack convergent validity. | As mentioned in the text, sampling for each of the exploratory and then confirmatory factor analyses was conducted separately from two separate samples. |
| 3- Insufficient Discriminant Validity: MSV exceeds AVE; yet, HTMT is below 0.85 to support this assertion. | The HTMT method offers greater precision in evaluating discriminant validity compared to the Fornell-Larcker criterion. Comprehensive explanations and supporting references on this topic have been thoroughly discussed and emphasized within the text. |
| 4- Doubtful Item Retention: Certain items exhibit low communalities (<0.2) and loadings (<0.3). More stringent cutoffs (such as >0.4) would be required. | Although Item S8 exhibited a factor loading of 0.405 and a communality of 0.254, it was retained due to its theoretical alignment with the Negative Self-Esteem factor and its contribution to the overall model fit. Following Hair et al. (2019), items with loadings≥0.30 were considered acceptable when supported by strong validity indices (RMSEA=0.049, CFI=0.981) and scale reliability (Cronbach’s α>0.8) |
| 5- Excessive reliance on fit indices: Although the model fit appears satisfactory, the significant χ² value (P<0.001) indicates potential inadequacies in the model's fit. | This concern is valid; however, the significance of this indicator is likely attributable to the large sample size used in the current study—a common and expected outcome. Consequently, additional fit indices were employed to assess the confirmatory factor analysis, as discussed elsewhere in the text. |
| 6- Cross-Cultural Issues: The RSES is typically one-dimensional; however, the Persian version comprises two components: PSE and NSE. Does prejudice exist in culture or translation? Not examined. | Considering that self-esteem is a culturally sensitive construct, conducting an exploratory factor analysis (EFA) was warranted. A comprehensive explanation for the use of both exploratory and confirmatory factor analyses is provided in the following comments. The divergence between the original scale’s unidimensional structure and the two-factor structure identified in the Persian version may be explained by the use of more advanced statistical techniques and cultural differences between the study populations. These issues are elaborated upon in the responses to the reviewers and the main manuscript. All recent revisions have been highlighted in yellow. |
| 7- Reporting Deficiencies: There is an absence of conflict of interest disclosure and no sample division for EFA/CFA validation.  Conclusion: The Persian RSES appears promising; nonetheless, sample bias, issues with factor analysis, and inadequate validity metrics undermine its reliability. | Thank you for your thoughtful feedback on our manuscript. We appreciate your time and effort in reviewing our work. Below, we address your concerns and provide revisions to strengthen the manuscript:  Conflict of Interest Disclosure: We have now included the following statement in the "Competing Interests" section:  "The authors declare that there are no conflicts of interest regarding the publication of this paper."  Sample Division for EFA/CFA: We have added the following text to the "Construct validity" subsection of the "Materials and methods" section to clarify the sample division:  “To achieve this goal, the entire dataset of 533 Iranian adults was randomly divided into two independent sets. The first independent and random dataset of 270 Iranian adults was used to conduct EFA, and the second independent and random dataset of 263 Iranian adults was used to conduct CFA.  This approach aligns with recommendations for rigorous psychometric evaluation, as it ensures that the factor structure derived from EFA is cross-validated in a separate sample during CFA, minimizing overfitting and enhancing generalizability (Brown, 2015; Watkins, 2018). Such separation is particularly critical when establishing a new cultural adaptation of a scale, as it strengthens confidence in the stability of the identified dimensions.”   - **Brown TA, editor Confirmatory factor analysis for applied research, 2nd ed. Confirmatory factor analysis for applied research, 2nd ed; 2015; New York, NY, US: The Guilford Press.** - **Watkins MW. Exploratory factor analysis: A guide to best practice. Journal of Black Psychology. 2018;44(3):219-46. doi: 10.1177/0095798418771807.** |
| **Reviewer 1** | **Response** |
| Thank you for the opportunity to review the manuscript, which is about Validity and reliability of the Rosenberg Self-Esteem Scale: A methodological study among the Iranian adult population  The content is very good buy I recommend some suggestions in order to improve it.  First, the paper should be revised by a native English speaker to refine the grammatical problems  1. Please attach the translation permission from the original author of the instrument. | The authors sincerely appreciate your positive feedback and valuable comments.  In addition to revising the entire manuscript text for compliance with native language grammar, permission for translating and psychometrically evaluating the Rosenberg Self-Esteem Scale (RSES) was uploaded as a supplementary file. |
| 2. Given that the scale was previously developed, why were face and content validity done quantitatively? What if the results recommended deleting item(s) before conducting a factor analysis? Is there any possibility to delete the item(s)? In this case, what were the benefits of conducting these time-consuming processes? | Since the translation and cultural adaptation from English to Persian have been redone, taking into account changes in the studied communities that could potentially impact the explanation of the concept of self-esteem, the face validity and content validity were reevaluated using both quantitative and qualitative approaches. This was necessary because the psychometric properties of the RSES were assessed in the general Iranian population, which differs statistically from the communities previously studied. Additionally, incomplete and contradictory reports from prior studies further emphasized the importance of a quantitative evaluation of the face validity and content validity of the Persian version of the RSES.  The authors' approach to items with IS, CVR, and I-CVI values below the cutoff point (1.5, 0.56, and 0.79, respectively) in terms of cultural relevance, as determined by the research team, involved a revision in both face validity and content validity stages, as mentioned in the manuscript text. If an item had IS, CVR, and I-CVI values below the cutoff point and did not align with the Iranian cultural context, it was removed. Subsequently, exploratory and confirmatory factor analyses were conducted on the scale with a reduced number of items.  Although all items had IS, CVR, and I-CVI values greater than the cutoff point, none of the items were eliminated in the aforementioned steps, and all items remained for subsequent analyses. |
| 3. Please explain why an Exploratory Factor Analysis (EFA) was conducted on a previously developed scale with well-determined factors. In such cases, as a validation process, conducting a Confirmatory Factor Analysis (CFA) to ensure the model's fitness is sufficient. Please justify the reason for conducting an EFA before a CFA. What was unclear that needed to be "explored"? | As noted in the discussion section, prior research has reported both one-factor and two-factor models of the RSES. In light of these inconsistencies, the research team performed an exploratory factor analysis in the present study to identify the underlying factor structure of the scale. |
| 4. In the construct validity section, specify exactly how many samples were included in the study for each of the stages of exploratory and confirmatory construct validity? | The exact number of samples to conduct each of the factor analysis approaches, both exploratory and confirmatory, was mentioned in the opening paragraph of the construct validity section of the materials and methods.  Changes are highlighted in yellow. |
| 5. Before conducting exploratory factor analysis, item analysis is performed to examine the correlation between each item's score and the scores of other items and the total score. Additionally, items that affect reliability are identified. Based on this section, the mean and standard deviation for the entire questionnaire and each item (including the highest and lowest scores) are reported. These details have not been reported here. | Thank you for your valuable feedback regarding the need for more detailed item analysis prior to conducting EFA. We appreciate your attention to methodological rigor and have carefully considered your suggestion.  To address this point, we have now included a comprehensive item analysis in the revised manuscript, reporting:  The mean and standard deviation for each item and the total scale  Item-total correlations  Reliability analysis (Cronbach's alpha if item deleted)  This additional analysis strengthens our psychometric evaluation and provides readers with a clearer understanding of the scale's properties at the item level. The new text has been added to the Materials and methods section (under "Construct validity"), and we have included appropriate citations to support this approach. |
| 6. Based on the suggestions of the expert panel during the qualitative content validity stage, some items were revised and modified…(Face and content validity…P10)...  Please specify which items were modified and in what way during the content validity process.  Were the items that were rewritten or modified during content validity adjustment changed because they did not achieve an adequate CVI score, or was there another reason for the changes? | Thank you for your valuable feedback.  Based on the suggestions of the expert panel during the qualitative content validity stage, items 1, 2, 3, 4, and 6 underwent minor changes in terms of grammar and word usage. It is important to note that the CVR, I-CVI, and MKS values for all items were excellent and above the threshold. Therefore, during the quantitative content validity stage, no items were edited or deleted, and all items were kept for further analysis.  These specifics are outlined in the content validity section of the results.  Changes are highlighted in yellow. |
| 7. In the results section, please mention the names of the extracted factors and indicate whether these items were the same in the original version and the translated version. Were their arrangement and order different or the same? This should also be explained in the discussion section. | The names of the factors in the EFA results table were mentioned in the original manuscript. They were also mentioned and highlighted in the text of the results section. In addition, the difference in the number of factors in the original and Persian versions of the RSES was discussed in the discussion section. New changes were highlighted. |
| 8. To mention what countries did this questionnaire | This study was conducted in Iran. We mentioned this in the methods and results sections. |
| 9. What is the reason for doing psychometrics in Iran. | Self-esteem is a concept deeply influenced by cultural and contextual factors. To ensure that a measure of such subjective concepts is suitable for a new cultural context, a careful and systematic process of translation and adaptation must be undertaken. We mentioned these explanations at the end of the introduction section and highlighted. |
| 10. In Table 1, the samples should be written separately. (EFA and CFA) | In Table 1, the demographic characteristics of the participants were presented separately for exploratory and confirmatory factor analysis.  Changes are highlighted in yellow. |
| 11. Why are the samples in separate exploration and confirmation steps with reference and reference؟ | Thank you for your valuable comment regarding the separation of samples for EFA and CFA factor analyses. We appreciate your attention to methodological rigor.  The division of the sample into two independent subsets (EFA: n=270; CFA: n=263) was intentional and aligns with best practices in psychometric validation studies. This approach ensures that:  Cross-Validation: The factor structure identified in EFA is independently tested in CFA, reducing the risk of overfitting and increasing the robustness of findings (Brown, 2015).   - **Brown TA, editor Confirmatory factor analysis for applied research, 2nd ed. Confirmatory factor analysis for applied research, 2nd ed; 2015; New York, NY, US: The Guilford Press.**   Methodological Rigor: Separate samples prevent capitalization on chance, providing more reliable evidence for the scale’s validity (Watkins, 2018).   - **Watkins MW. Exploratory factor analysis: A guide to best practice. Journal of Black Psychology. 2018;44(3):219-46. doi: 10.1177/0095798418771807.**   We have added a clarification to the Materials and methods section to explicitly justify this design choice. We hope this addresses your concern and enhances the transparency of our methodology. |
| 12. The discussion could be expanded to address potential limitations in the cultural adaptation process. For example, how cultural differences might influence responses beyond translation accuracy. | Although the Persian version of the RSES demonstrated acceptable psychometric properties, it is important to acknowledge certain limitations in the cultural adaptation process. Cultural adaptation extends beyond mere translation accuracy, encompassing deeper cultural, social, and psychological factors that shape how respondents interpret the concept of self-esteem. In the Iranian context, prevailing cultural norms may influence individuals’ responses and, consequently, affect the scale’s validity. Future research should adopt more comprehensive cultural adaptation approaches, incorporating qualitative methods and the involvement of cultural experts, to more accurately capture the nuanced expression of self-esteem within Iranian culture and enhance the scale’s applicability.  We add these explanations at the end of the discussion section. |
| **Reviewer 2** | **Response** |
| Thanks for inviting me to review this study. here there are some recommendations for authors:  Why measuring self-esteem in adults is important? | Thank you for your valuable comment.  The importance of measuring self-esteem in adults was discussed in the opening paragraph of the introduction.  Changes are highlighted in yellow. |
| What is the problem with self-esteem in your country? | In Iran, self-esteem is closely linked to spirituality and is considered essential for moral behavior and mental health. Self-esteem is valued as a cultural priority, with the belief that each person is uniquely valued by God. Therefore, before low self-esteem is considered a problem, its promotion is a social value. We have presented these points specific to Iranian culture in the introduction. |
| Your reasons for conducting a new study were not convincing. Instead of emphasizing whether or not confirmatory factor analysis was conducted, you should focus on the study population because most of the studies were on students. | The final paragraph of the introduction emphasized the importance of conducting research, particularly focusing on the statistical population under study.  Changes are highlighted in yellow. |
| Move the study population section to the beginning of the method after “Design and purpose”. | It’s done. |
| Reference 21 should be corrected; the link is not active. | Thank you. We replaced this reference with appropriate reference. |
| Since nowadays many studies are published in the field of psychometrics and readers are familiar with the basic concepts, it would be better to write the translation section, face, and content validity, more concisely. | Thank you for your suggestion. We summarized the explanations of face and content validity. New changes are highlighted. |
| what was the Data collection procedure? Online? Paper? what were the demographic variables? Where do you find the samples? what was the response rate? | The researchers conducted a survey by sharing a link to the online data collection form on social media platforms and various public information channels throughout the city. The demographic variables collected included gender, marital status, educational level, employment status, and age. According to the database records, 540 participants completed the online form. However, seven respondents were excluded from the analysis because they did not complete the RSES-specific section. As a result, the effective response rate was estimated at 98.7%. We added new information in the text and highlighted. |
| How do you select 30 Iranian adults for ICC? | Thirty participants were conveniently selected from the community to take part in the RSES test-retest evaluation, ensuring a diverse representation of gender, education, occupation, age, and marital status. No specific guidelines were established for sampling in the test-retest phase. |
| Please report gender in the text of results, also the range for age. | The gender and age range of the participants were reported in the opening paragraph of the results.  Changes are highlighted in yellow. |
| What was the method of keeping items in CFA? Because you keep item S8 with 0.4 in CFA. | Thank you for your insightful comment regarding the retention of items in the CFA. We appreciate your attention to methodological rigor.  In our study, item S8 (with a factor loading of 0.4) was retained based on the following considerations:  Theoretical Alignment: The item aligns with the Negative Self-Esteem (NSE) factor, as defined in prior research (e.g., Rosenberg, 1965; Syropoulou et al., 2021).   - **Rosenberg M. The measurement of self-esteem, society and the adolescent self-image. Princeton. 1965:16-36.** - **Syropoulou A, Vernadakis N, Papastergiou M, Kourtessis T. Psychometric evaluation of the Rosenberg Self-Esteem Scale in primary school students with mild intellectual disability: First evidence. Research in Developmental Disabilities. 2021;114:103964. Epub 2021/05/22. doi: 10.1016/j.ridd.2021.103964. PubMed PMID: 34020410.**   Communality and Model Fit: Despite the modest loading, the item's communality (0.254) exceeded the recommended threshold of 0.2 (Hair et al., 2019), and its inclusion improved overall model fit indices (RMSEA=0.049, CFI=0.981).   - **Hair JF, Black WC, Babin BJ, Anderson RE. Multivariate Data Analysis: Cengage; 2019.**   Comparative Validity: Previous studies (e.g., Supple et al., 2013) have retained items with similar loadings when they contribute to scale consistency and theoretical coherence.   - **Supple AJ, Su J, Plunkett SW, Peterson GW, Bush KR. Factor structure of the Rosenberg self-esteem scale. Journal of Cross-Cultural Psychology. 2013;44(5):748-64**   We acknowledge that lower loadings can raise concerns, but given the strong psychometric properties of the overall scale, we deemed its retention justified. Below, we have added a clarification to the manuscript to address this point explicitly. |
| Please provide 95%CI for reliability indices. | The 95% confidence interval for the intraclass correlation coefficient (ICC) is provided in Table 3.  Changes are highlighted in yellow. |
| Please provide a table for the Heterotrait-monotrait ratio of correlations (HTMT) | Since this table contains only a single value, we have reported it directly in the text to avoid presenting an excessive number of tables. We provided the tables below for your consideration: \| PSE \| NSE; PSE \| \| 0.769; NSE \| 0.769 \| **HTMT Analysis** |
