# Supplementary material for: Validation of the Rosenberg Self-Esteem Scale among the Iranian adult population: A cross-sectional study
Source: PLoS One. 2025 Dec 15;20(12):e0336969. doi: 10.1371/journal.pone.0336969 (PMC12799268; doi:10.1371/journal.pone.0336969)
Supplement: Attachment — Submitted filename: Response to reviewers _ Version 02.docx [file pone.0336969.s005.docx]

**Dear Editor, PLOS One,**

Thank you for carefully reviewing and considering our manuscript, titled **"Validation of the Rosenberg Self-Esteem Scale among the Iranian adult population: A cross-sectional study"**, submitted to ***PLOS One***.

We sincerely appreciate the thoughtful comments and suggestions provided by the reviewers, which have greatly enhanced the quality and depth of our manuscript. In the revised version, all changes made in response to the feedback have been highlighted. Additionally, detailed responses to each comment are provided below.

Please feel free to contact me if you have any questions or need further clarification. We look forward to your response regarding the manuscript.

Thank you once again for your time and support.

Sincerely,

Corresponding author

| **Journal Requirements** | **Response** |
| --- | --- |
| If the reviewer comments include a recommendation to cite specific previously published works, please review and evaluate these publications to determine whether they are relevant and should be cited. There is no requirement to cite these works unless the editor has indicated otherwise. |  |
| **Additional Editor Comments** | **Response** |
| Exhaustive Review Report  Manuscript Title:  Validity and reliability of the Rosenberg Self-Esteem Scale: A methodological study among the Iranian adult population  Title  • The title accurately reflects the study’s focus but would benefit from specifying the cross-sectional validation design to better align with methodological expectations for psychometric studies. | Thank you very much.  We revised the title as below:  *Validation of the Rosenberg Self-Esteem Scale among the Iranian adult population: A cross-sectional study* |
| Abstract  • The abstract omits essential psychometric indicators, such as reliability coefficients (e.g., Cronbach’s alpha, composite reliability) and model fit indices (e.g., RMSEA, CFI), which are standard in scale validation studies.  • It does not mention adherence to STROBE guidelines or detail the statistical approach for ordinal data. | Thank you for your valuable feedback.  We provided essential psychometric indicators in the abstract and highlighted.  In addition to uploading the STROBE checklist as a supplementary file, instructions on how to handle ordinal data were also included. |
| Introduction  Literature Review  • Previous validations of the RSES (e.g., Kielkiewicz et al., Tulachan et al.) are cited without reporting key psychometric metrics (e.g., factor structure, reliability coefficients, fit indices), limiting comparative and interpretative value.  • The rationale for distinguishing “students” from “adults” is unclear and unsupported, as students fall within the adult population. | We appreciate these suggestions. Literature reviews were improved by adding additional information. Also, we clarified our rationale about distinguishing “students” from “adults” to support conducting the present study. |
| Cultural Adaptation Rationale  • The manuscript refers to “cultural adaptation,” but only describes translation procedures. There is no evidence of conceptual or cross-cultural equivalence testing, cognitive interviews, or qualitative item evaluation supporting adaptation claims. | Thank you for your constructive feedback.  First, we separated the translation stages and identified the members of each stage to enhance the readers' understanding. Next, we included additional items related to cultural adaptation, such as assessing conceptual or cross-cultural equivalence by a team of experts, and conducting cognitive interviews to evaluate the items for comprehension, acceptability, and emotional impact. |
| **Methods**  ***Participants***  • Inclusion/exclusion criteria (e.g., age range, educational background, psychiatric conditions) are not specified. | Thanks. We added the accurate inclusion and exclusion criteria in this study. |
| • Sampling imbalance is unaddressed: students constitute 42.8% of the sample and divorced/widowed participants are underrepresented. | We emphasized this limitation at the end of the Discussion section. We turned this sentence red.  *“A considerable proportion of the participants in this study were young, single students from Shahroud County in northeastern Iran, which may restrict the generalizability of the findings to the wider population.”* |
| • The dataset split into EFA (n=270) and CFA (n=263) subsamples lacks details on:  o Randomization method (e.g., simple vs. stratified)  o Verification of demographic equivalence across subsamples  o Software or tools used for splitting | Thank you for your valuable and insightful comments. We sincerely appreciate you highlighting the need for greater detail regarding the dataset split. In response, we have revised the “Construct validity” subsection in the Materials and methods to explicitly state:  The randomization method (simple random sampling without replacement via SPSS).  The software used for splitting the data (SPSS version 26).  The statistical tests performed to verify the demographic equivalence of the two subsamples (independent t-tests and chi-square), confirming no significant differences were found. |
| ***Test-Retest Sample***  • While demographic details were used to select the 30 participants in the test-retest subsample, these data are missing for the main sample (N=533) and the EFA/CFA subsamples, limiting transparency on representativeness. | In the earlier version of the manuscript, detailed information for the main sample (N=533) as well as the EFA and CFA subsamples was presented in Table 1, with a summary also provided in the text. Please see the highlighted sections in red in Table 1 and the accompanying explanations. |
| ***RSES Translation***  • No justification is provided for retranslating the RSES despite existing validated Persian versions. | Detailed and well-supported justifications, grounded in previous literature and relevant critiques, are provided in the Introduction. Therefore, the response to this comment is addressed within the Introduction section rather than the Materials and methods section. |
| • The composition and expertise of expert panels for translation and content validity are insufficiently detailed:  o Unclear if panels overlapped  o No description of disagreement resolution or panel qualifications  o No mention of how panel size impacted CVR/I-CVI interpretation (e.g., Lawshe’s critical values) | The following were included in the “Content Validity” subsection of the Materials and Methods:  (1) Composition of expert panels for assessing content validity.  (2) Non-overlapping panels, meaning that experts in the first panel assessed the necessity of items to calculate the Content Validity Ratio (CVR), while experts in the second panel assessed the relevance of items to calculate the Content Validity Index (CVI).  (3) How to calculate the Modified Kappa (MKS) statistic to minimize chance agreement among experts.  (4) How to determine the minimum CVR value based on the Lawshe table. |
| ***Statistical Analysis***  Ordinal Data Violations  • Maximum Likelihood Estimation (MLE) was used for EFA and CFA despite the ordinal Likert-type responses (1–5), violating assumptions of continuity and normality. | Thank you for your valuable and insightful comments regarding the appropriate statistical treatment of ordinal data. We sincerely appreciate you highlighting this important methodological consideration. In response to your feedback, we have revised the “Construct validity” subsection in the Materials and methods to explicitly state:  The use of polychoric correlations as the input matrix for factor analysis  The employment of robust weighted least squares (WLSMV) estimation for both EFA and CFA  The rationale for using ordinal-appropriate estimation methods given the Likert-type nature of our data  The use of appropriate measures to examine underlying multivariate normality assumptions |
| • No rational or empirical evidence supports treating ordinal data as interval-level. |  |
| • No ordinal-appropriate estimation methods (e.g., polychoric correlations, WLSMV) were employed. |  |
| • The skewness/kurtosis thresholds (-3 to +3 and -7 to +7) cited are inappropriate for ordinal data. |  |
| ***Outlier Detection***  • Mahalanobis distance was used to identify univariate and multivariate outliers but assumes continuous, normally distributed variables—this is inappropriate for ordinal Likert data. | Thank you for this crucial observation. You are absolutely correct that AMOS does not support the WLSMV estimator, which is necessary for robust CFA with ordinal data. we have revised the “Construct validity” subsection to accurately reflect the software used. The analyses were, in fact, conducted using JASP 0.19.3.0 and its WLSMV estimator, which is specifically designed for ordinal data. |
| • The manuscript does not specify how many cases were flagged or excluded, nor any sensitivity analyses assessing their impact. |  |
| • Alternative outlier detection methods suited to ordinal data (e.g., response pattern analysis) were not considered. |  |
| ***Software Limitations***  • AMOS was used for CFA but lacks estimators robust to ordinal data (e.g., DWLS, WLSMV). |  |
| • ML estimation with ordinal indicators can bias factor loadings, underestimate standard errors, and inflate model fit indices. |  |
| **Results**  ***EFA and CFA***  • Item S8 was retained despite a low factor loading (0.405) and communality (h²=0.254) without justification. | In the previous version of the manuscript, we clearly specified the critical values for factor loadings and communalities as follows: ***“It is important to note that items with a communality of less than 0.2 and a factor loading of less than 0.3 were eliminated.”*** Based on these criteria, Item 8 was retained. |
| • CFA fit indices (e.g., CFI=0.981, RMSEA=0.049) may be inflated due to improper treatment of ordinal data as continuous. | Thank you for your insightful comments. We have now conducted a sensitivity analysis comparing WLSMV and ML estimators, confirming the robustness of our factor structure. We have also clarified the convergent validity by referencing both CR>AVE and standardized factor loadings>0.5. These changes have been incorporated into the manuscript. |
| • No sensitivity analysis comparing ordinal-appropriate versus continuous-variable assumptions was performed. |  |
| ***Validity and Reliability***  • Convergent validity accepted AVE values below 0.5 solely because CR>AVE, contrary to Fornell-Larcker criteria. |  |
| • HTMT ratio (0.769) approaches the 0.85 discriminant validity threshold but is not discussed. | The results showed an HTMT value of 0.769, indicating an acceptable level of discriminant validity between the two factors. Although this value approaches the critical threshold, it still statistically supports adequate discriminant validity. We discussed in the text and highlighted. |
| • Crucially, no results for the total RSES score are provided — no internal consistency, test-retest reliability, or descriptive statistics. Given the RSES’s widespread use as a unidimensional scale, this omission critically limits the study’s applicability. | Thank you for your constructive feedback.  The mean (SD) scores for total self-esteem, positive self-esteem (PSE), and negative self-esteem (NSE) of the participating adults were reported. Additionally, the internal consistency and test-retest reliability of the entire scale were also calculated. |
| **Discussion**  ***Factor Structure***  • The discussion focuses on a two-factor model but neglects to explain why a single-factor model, supported in many contexts, was not viable or retained. | At the end of the discussion of the factor structure results, possible reasons for obtaining a two-factor structure and possible causes for obtaining findings different from previous literature (which generally reported a single-factor structure) were presented and highlighted. |
| ***Cultural Context***  • There is no consideration of Iranian cultural factors (e.g., modesty bias, social desirability) that could influence responses, particularly to negatively worded items. | We have made the necessary revisions and included additional explanations in accordance with the previous reviewer’s comment. |
| ***Generalizability***  • The manuscript overstates generalizability without addressing:  o Overrepresentation of students (42.8%)  o Sampling restricted to Shahroud County (urban bias) | We were accurate discussed about these limitations at the end of the Discussion section. We made these limitations in red. |
| Comparative Interpretation  • Comparisons with prior studies (e.g., Mayordomo et al.) lack quantitative benchmarking (e.g., fit indices, reliability coefficients). | Quantitative measures, such as goodness of fit and reliability coefficients, were included in the comparisons. |
| **Conclusions**  • The claim that the Persian RSES is “valid and reliable” is premature due to:  o Fundamental violations in the treatment of ordinal data  o Incomplete reporting of cultural adaptation processes  o Sampling biases limiting representativeness  o Absence of psychometric data for the total scale score | In the first step, we discussed how to handle ordinal data. In the second step, we outlined the translation and cultural adaptation process in the relevant subsection. In the third step, we addressed sampling biases in both the "Limitations, strengths, and suggestions" subsection and the Conclusion section. In the fourth step, we calculated psychometric indices for the total score of the scale.  The Conclusion section was also revised accordingly:  *The results of this study demonstrate that the Persian version of the RSES has good validity and reliability for measuring self-esteem in Iranian adults. However, caution should be taken when applying it to married and non-student adults, as the majority of participants in this study were single and student adults.* |
| **Ethics and Transparency**  • Verbal informed consent procedures are briefly mentioned but lack detail regarding documentation or verification.  • No data availability statement is provided.  • No IRB exemption or approval details for data restrictions are included.  • The manuscript does not explicitly state adherence to STROBE guidelines, and key items (e.g., handling of ordinal data, randomization protocol) are unreported. | • Thank you very much. We write inconsistent. So, we made clarification in the text about informed consent. We received only written informed consent due to online nature of data sampling.  • The data availability statement has been provided.  • We have no data restrictions. All of approval procedure and approval code were provided in the “Ethical considerations” subsection.  • In addition to uploading the STROBE checklist as a supplementary file, instructions on how to handle ordinal data and the randomization protocol were also reported. |
| **Overall Assessment**  While the study’s design—especially the split-sample approach for EFA and CFA—is methodologically sound in principle, execution and reporting weaknesses undermine its contribution:  • Statistical Misapplication: Use of ML-based EFA/CFA and Mahalanobis distance for ordinal Likert data without justification or robustness checks. | We sincerely thank the reviewer for this insightful comment. We acknowledge the concern regarding the use of maximum likelihood (ML) estimation and Mahalanobis distance with ordinal data.  In response, we have thoroughly revised our analytical approach. The manuscript now explicitly states the use of the robust weighted least squares (WLSMV) estimator, which is specifically recommended for ordinal data, for both our exploratory and confirmatory factor analyses. We have also removed any mention of Mahalanobis distance and replaced our outlier detection method with a detailed description of response pattern analysis suitable for categorical data.  A sensitivity analysis confirmed that the factor structure remained consistent across estimators, supporting the robustness of our findings. These revisions are detailed in the “Construct Validity” subsection. |
| • Reporting Deficiencies: Incomplete documentation of cultural adaptation, sampling procedures, expert panels, and outlier management. | Thank you very much. We added additional explanations and information to cover mentioned gaps. New added information is highlighted. |
| • Transparency Gaps: Lack of data sharing statements, IRB documentation, and STROBE adherence. | The requested items have been added. |
| Key Recommendations for Revision  • Report demographic characteristics for the full sample and EFA/CFA subsamples to ensure representativeness. | In the earlier version of the manuscript, detailed information for the main sample (N=533), as well as for the EFA and CFA subsamples, was presented in Table 1, with a corresponding summary provided in the text. Please refer to the highlighted sections in red within Table 1 and the related explanations. |
| • Justify and clearly describe outlier detection methods appropriate for ordinal data and report their handling and impact. | Based on the reviewer’s comment, we have revised the manuscript to include a detailed description of the outlier detection methods used for ordinal data. The following paragraph was added to the Materials and methods section under “Construct validity”:  *For outlier detection in ordinal data, we employed a combination of methods suitable for categorical variables. We conducted response pattern analysis to identify inconsistent or atypical response patterns across the 10 items. Additionally, we used the mean and standard deviation of each item to flag cases with extreme values (e.g., all items rated as 1 or all as 4). Through this process, 7 cases (1.3% of the total sample) were identified as outliers based on their atypical response patterns and were excluded from subsequent analyses. A sensitivity analysis was conducted by comparing the factor structure with and without these outliers, which showed no substantial differences in factor loadings or model fit indices, confirming the robustness of our results.* |
| • Include psychometric results for the total RSES score (internal consistency, test-retest reliability, descriptive statistics). | Thank you for your valuable feedback.  Cronbach's alpha and McDonald's omega coefficients, along with the intraclass correlation coefficient (ICC), were calculated to evaluate the **internal consistency** and **test-retest reliability** of the entire scale, respectively. The mean (SD) of the overall self-esteem scores of the participating adults was also reported. |
| • Consider analyzing a unidimensional or higher-order CFA model supporting the total score’s validity. | Thank you for this suggestion. As the primary aim of our study was to validate the two-factor structure (PSE and NSE) derived from the Iranian context, and since discriminant validity between these factors was established (HTMT = 0.769), we focused our analysis on confirming this model. We therefore did not test a unidimensional or higher-order CFA model in this validation study. |
| • Provide full details of the expert panels’ composition, qualifications, and item evaluation process. | Thank you for your constructive feedback.  Full details were provided regarding the composition of the expert panels, including the number of experts in each field. Additionally, the method for calculating various indices such as CVR, CVI, and MKS to quantitatively assess content validity was detailed. |
| • Adjust statistical methods for ordinal data, potentially employing polychoric correlations and robust CFA estimators. | Thank you for this crucial comment. We have now revised our statistical approach to fully accommodate the ordinal nature of the data. The manuscript has been updated to employ polychoric correlations and the robust weighted least squares (WLSMV) estimator for both EFA and CFA, as recommended. |
| • Detail informed consent procedures and provide data availability and ethical approval statements. | The requested items have been added. |
| • Align the manuscript’s title and abstract with the scope and findings of the analyses performed. | The title and abstract of the manuscript accurately reflected the scope and findings of the study. |
| **Reviewer 3** | **Response** |
| Thank you for submitting your revised manuscript, "Validity and reliability of the Rosenberg Self-Esteem Scale: A methodological study among the Iranian adult population" (PONE-D-25-07036R1). The authors have made significant efforts to address the concerns raised by the reviewers, leading to a much-improved manuscript.  General Comments:  The manuscript is well-written and presented in an intelligible fashion, adhering to standard English. The revisions have substantially enhanced the clarity and rigor of the methodology and discussion. | Thank you so much. |
| **Technical Soundness and Statistical Analysis:**  The manuscript describes a technically sound piece of scientific research. The experiments appear to have been conducted rigorously, with appropriate controls and sample sizes, particularly with the clarified separation of samples for EFA and CFA. The statistical analysis has been performed appropriately and rigorously, with the authors providing clear justifications for their methodological choices, such as the retention of Item S8 and the use of the HTMT method for discriminant validity. The conclusions drawn are well-supported by the data presented in the revised manuscript. | Thank you for your positive viewpoints. |
| **Data Availability:**  While the authors state that "All relevant data are within the manuscript and its Supporting Information files," it appears that the raw data underlying the findings are not fully available in a format that allows for replication of all study findings. To comply with the PLOS Data Policy, authors are required to make the minimal data set publicly available without restriction. This includes, for example, the individual data points behind means, medians, and variance measures reported in the tables and text. Please ensure that all raw data points required to replicate the results of your study are either uploaded as Supporting Information files or deposited to a stable, public repository, with the relevant URLs, DOIs, or accession numbers provided. If there are ethical or legal restrictions on sharing a de-identified data set, these must be explained in detail, along with contact information for a data access committee or institutional body.  Overall, the manuscript is a strong contribution to the literature, and addressing the data availability concern will further enhance its completeness and transparency. | The data supporting the results of this study contain potentially personally identifying and sensitive information about the participants. Therefore, this data cannot be made publicly available according to the policies and regulations of the Research Ethics Committee of Shahroud University of Medical Sciences. The ethical approval of this study also explicitly prohibits public sharing of the data to protect the confidentiality and privacy of the participants. However, the data will be available upon reasonable request from the corresponding author via email ([abbasi_msn@yahoo.com](mailto:abbasi_msn@yahoo.com)) or from the Vice President for Research and Technology of this university via email ([vcr@shmu.ac.ir](mailto:vcr@shmu.ac.ir)) to researchers who meet the criteria for access to confidential data.   1. **Ali Abbasi (Corresponding Author)**   E-mail: [abbasi_msn@yahoo.com](mailto:abbasi_msn@yahoo.com)   2. **Vice President for Research and Technology of Shahroud University of Medical Sciences**   E-mail: [vcr@shmu.ac.ir](mailto:vcr@shmu.ac.ir)  In compliance with the local policies of Shahroud University of Medical Sciences, the data availability statement has been revised as follows:  *The data supporting the results of this study contain potentially personally identifying and sensitive information about the participants. Therefore, this data cannot be made publicly available according to the policies and regulations of the Research Ethics* *Committee of Shahroud University of Medical Sciences. The ethical approval of this study also explicitly prohibits public sharing of the data to protect the confidentiality and privacy of the participants. However, the data will be available upon reasonable request from the corresponding author via email (*[*abbasi_msn@yahoo.com*](mailto:abbasi_msn@yahoo.com)*) or from the Vice President for Research and Technology of this university via email (*[*vcr@shmu.ac.ir*](mailto:vcr@shmu.ac.ir)*) to researchers who meet the criteria for access to confidential data.* |
| **Reviewer 4** | **Response** |
| While the language is intelligible, it is suggested to further improve to standard English. | Thank you for your valuable feedback.  Further grammatical corrections have been made to bring the manuscript up to standard English. |
| Write details of how sample size was calculated, even if it has been referenced. | More details were provided on how to calculate the sample size. |
| Write setting and data collection in detail process of how participants were approached, in further detail. | We provided more information about study setting and highlighted in yellow. In the previous version we provided accurate information about data collection process. We made these explanations in red. |
| How did you make sure that only Shahroud city participants fill online forms? Since online links can be accessed by Persian speaking people anywhere in the country, or even outside? | Invitations to participate in the study were distributed through channels dedicated to local events and content in Shahroud. To ensure that participants were residents of Shahroud, individuals were asked to confirm their place of residence via a self-report question before accessing the online questionnaire.  We added these explanations in the text. |
| Please mention convenience sampling and online data collection in limitation section in your study limitations. | Thank you very much. We added these limitations in the text and highlighted. |
