# Supplementary material for: Validation of the Rosenberg Self-Esteem Scale among the Iranian adult population: A cross-sectional study
Source: PLoS One. 2025 Dec 15;20(12):e0336969. doi: 10.1371/journal.pone.0336969 (PMC12799268; doi:10.1371/journal.pone.0336969)
Supplement: Attachment — Submitted filename: Response_to_reviewers_auresp_3.docx [file pone.0336969.s006.docx]

**Dear Editor, PLOS One,**

Thank you for carefully reviewing and considering our manuscript, titled **"Validation of the Rosenberg Self-Esteem Scale among the Iranian adult population: A cross-sectional study"**, submitted to ***PLOS One***.

We sincerely appreciate the thoughtful comments and suggestions provided by the reviewers, which have greatly enhanced the quality and depth of our manuscript. In the revised version, all changes made in response to the feedback have been highlighted. Additionally, detailed responses to each comment are provided below.

Please feel free to contact me if you have any questions or need further clarification. We look forward to your response regarding the manuscript.

Thank you once again for your time and support.

Sincerely,

Corresponding author

| **Journal Requirements** | **Response** |
| --- | --- |
| If the reviewer comments include a recommendation to cite specific previously published works, please review and evaluate these publications to determine whether they are relevant and should be cited. There is no requirement to cite these works unless the editor has indicated otherwise.  Please review your reference list to ensure that it is complete and correct. If you have cited papers that have been retracted, please include the rationale for doing so in the manuscript text, or remove these references and replace them with relevant current references. Any changes to the reference list should be mentioned in the rebuttal letter that accompanies your revised manuscript. If you need to cite a retracted article, indicate the article’s retracted status in the References list and also include a citation and full reference for the retraction notice. | We have reviewed all suggested references and ensured that the reference list is accurate, complete, and free of retracted articles. Any necessary updates or replacements have been made and are reflected in the revised manuscript. |
| **Reviewer 5** | **Response** |
| Reviewer #5: Thank you for the opportunity to review the manuscript "Validity and reliability of the Rosenberg Self-Esteem Scale: A methodological study among the Iranian adult population". I agree with reviewer 3, that the authors have made significant efforts to address the concerns raised by the reviewers, leading to a much-improved manuscript. The methodological approach of combined exploratory and confirmatory is now well explained in the Introduction and embedded in previous research findings. The translation and initial validation process of the Persian version is transparently described in the Methods section. The Methods section also includes a thorough overview of the analyses conducted to establish the scale’s validity. | Thank you so much. |
| ***General remarks:***  Please check the manuscript for adherence to APA 7th guidelines on reporting statistics (e.g. statistics in italic). | We checked the manuscript and modified the text based on the APA 7th guidelines on reporting statistics. New changes are highlighted. |
| ***Methods & Results:***  Based on a simulation study, Golino et al. (2021) advised to interpret fit indices as relative rather than absolute since fit indices are influenced for instance by sample size, number of items or correlation between latent factors. They conclude that one cut-off value can hardly consider all these factors and model fit indices should rather be interpreted as relative. I therefore suggest to add a 1-factor self-esteem model to the confirmatory factor analysis and compare the model fit of the 1- and 2-factor model to further underline the superiority of the 2-factor model. | A single-factor self-esteem model was added to the confirmatory factor analysis and the fit of the single-factor and two-factor models was compared. According to the results, the superiority of the two-factor model was highlighted. New changes were highlighted. |
| ***Discussion:***  How did you calculate the total self-esteem score? Were the negatively worded items reverse-coded? I would also suggest to add a recommendation for users on how to calculate and use the self-esteem scores (e.g. should users calculate separate sum scores for each scale or can they also calculate a total sum score if the negative items are reverse-coded?). | Thank you for your valuable recommendation. We clarified the text based on your concern:  *The Persian version of the self-esteem scale includes 10 items assessing two dimensions: positive self-esteem (PSE; items 1, 2, 4, 6, and 7) and negative self-esteem (NSE; items 3, 5, 8, 9, and 10). Each item is rated on a four-point scale ranging from 1 (“I completely disagree”) to 4 (“I completely agree”). The negatively worded items (3, 5, 8, 9, and 10) were reverse-coded before computing the total score. A total self-esteem score can be obtained by summing all items after reverse-coding, with higher scores indicating greater overall self-esteem. Researchers may also calculate separate subscale scores for PSE and NSE when a more differentiated assessment of self-esteem dimensions is desired.* |
| The authors have thoroughly assessed the construct validity using different statistical approaches. I would suggest to add a suggestion for future research in the Discussion how the convergent and discriminant validity of the Persian version could be assessed with regard to similar or discrepant constructs (e.g. assessing the correlation with depressive symptoms for discriminant validity). I think that the authors’ insight on which constructs would be relevant to further assess the validity of the two self-esteem scales of the Persian version would be valuable.  I hope that my comments are understandable and can contribute to refining the manuscript. | Thank you for your positive suggestion. We add this recommendation for future studies at the end of the discussion section and highlighted. |
