# Supplementary material for: Validation of the Rosenberg Self-Esteem Scale among the Iranian adult population: A cross-sectional study
Source: PLoS One. 2025 Dec 15;20(12):e0336969. doi: 10.1371/journal.pone.0336969 (PMC12799268; doi:10.1371/journal.pone.0336969)
Supplement: Attachment — Submitted filename: Reviewer comments.docx [file pone.0336969.s004.docx]

Thank you for the opportunity to review the manuscript "Validity and reliability of the Rosenberg Self-Esteem Scale: A methodological study among the Iranian adult population". I agree with reviewer 3, that the authors have made significant efforts to address the concerns raised by the reviewers, leading to a much-improved manuscript. The methodological approach of combined exploratory and confirmatory is now well explained in the Introduction and embedded in previous research findings. The translation and initial validation process of the Persian version is transparently described in the Methods section. The Methods section also includes a thorough overview of the analyses conducted to establish the scale’s validity.

General remarks:

Please check the manuscript for adherence to APA 7^th^ guidelines on reporting statistics (e.g. statistics in italic).

Methods & Results:

Based on a simulation study, Golino et al. (2021) advised to interpret fit indices as relative rather than absolute since fit indices are influenced for instance by sample size, number of items or correlation between latent factors. They conclude that one cut-off value can hardly consider all these factors and model fit indices should rather be interpreted as relative. I therefore suggest to add a 1-factor self-esteem model to the confirmatory factor analysis and compare the model fit of the 1- and 2-factor model to further underline the superiority of the 2-factor model.

Discussion:

How did you calculate the total self-esteem score? Were the negatively worded items reverse-coded? I would also suggest to add a recommendation for users on how to calculate and use the self-esteem scores (e.g. should users calculate separate sum scores for each scale or can they also calculate a total sum score if the negative items are reverse-coded?).

The authors have thoroughly assessed the construct validity using different statistical approaches. I would suggest to add a suggestion for future research in the Discussion how the convergent and discriminant validity of the Persian version could be assessed with regard to similar or discrepant constructs (e.g. assessing the correlation with depressive symptoms for discriminant validity). I think that the authors’ insight on which constructs would be relevant to further assess the validity of the two self-esteem scales of the Persian version would be valuable.

I hope that my comments are understandable and can contribute to refining the manuscript.
